# The association of timing of repeat cesarean with outcomes among a cohort of Guatemalan women with a history of prior cesarean birth

**DOI:** 10.1186/s12884-021-04000-3

**Published:** 2021-07-20

**Authors:** Margo S. Harrison, Ana Garces, Lester Figueroa, Jamie Westcott, Michael Hambidge, Nancy F. Krebs

**Affiliations:** 1 University of Colorado School of Medicine Anschutz Medical Campus, Mail Stop B198-2, Academic Office 1, 12631 E. 17th Avenue, Rm 4211, Aurora, CO 80045 USA; 2 Institute of Nutrition of Central America and Panama, Guatemala City, Guatemala

**Keywords:** Mode of delivery after cesarean, Guatemala, Pre-labor cesarean, Intrapartum cesarean

## Abstract

**Background:**

The objective of this analysis was to observe whether maternal and perinatal/neonatal outcomes of birth vary by timing of repeat cesarean among women with a history of one prior cesarean birth in a Guatemalan cohort.

**Methods:**

This secondary analysis was conducted using data from a prospective study conducted in communities in Chimaltenango, Guatemala through the Global Network for Women’s and Children’s Health Research.

**Results:**

Between January 2017 and April 2020, 26,465 women delivered; 3,143 (11.9%) of those women had a singleton gestation and a history of prior cesarean delivery. 2,210 (79.9%) women with a history of prior cesarean birth had data available on mode of delivery and gave birth by repeat cesarean; 1312 (59.4%) were pre-labor cesareans while 896 (40.5%) were intrapartum cesarean births. Risk factors associated with an increased risk of intrapartum cesarean birth included hospital delivery as compared to “other” location (ARR 1.6 [1.2,2.1]) and dysfunctional labor (ARR 1.6 [1.4,1.9]). Variables associated with a reduced risk of intrapartum cesarean birth were hypertensive disease (ARR 0.7 [0.6,0.9]), schooling (ARR 0.9 [0.8,0.9]), and increasing age, which was associated with a very slight reduction in the outcome (ARR 0.99 [0.98,0.99]). Maternal and neonatal outcomes did not vary by type of cesarean birth.

**Conclusion:**

Outcomes of cesarean birth do not seem to vary by timing of repeat cesarean birth, with hypertensive disease increasing the likelihood of pre-labor cesarean. This information might be useful in counseling women that outcomes after failed trial of labor do not appear worse than those after pre-labor cesarean birth.

## Introduction

The World Health Organization (WHO) has recommended use of the Robson Classification for Cesarean Birth to observe subgroups contributing to cesarean birth rates within and across facilities and countries, over time [1]. When the classification system was applied to a large Guatemalan cohort, it was noted that the subgroup with the highest cesarean birth rate was multiparous women with a history of a prior uterine scar and a single, cephalic, term pregnancy, at 25.7% of all births in this cohort [2]. As cesarean birth rates increase globally, so does elective repeat cesarean birth; it is often the case that women with a history of prior cesarean account for one of the largest groups contributing to rising cesarean birth rates [3].

Women with a history of prior cesarean birth can elect to undergo repeat cesarean birth or pursue a trial of labor after cesarean [4]. Is it estimated that about 60 – 80% of women would be able to achieve vaginal birth after if they attempt trial of labor, but given the small risk of uterine rupture (< 1%), many women instead opt for elective repeat cesarean birth [4]. Repeat cesarean birth can occur electively prior to the onset of labor, or once labor has begun. Prior research has suggested that cesarean birth performed prior to the onset of labor can result in reduced maternal and perinatal morbidity and mortality [5]. The objective of this study was to observe characteristics associated with women who underwent pre-labor cesarean versus intrapartum repeat cesarean birth in a large Guatemalan cohort, as well as variability in maternal and perinatal outcomes by timing of the repeat cesarean birth. This cohort is compared to women who underwent vaginal birth after cesarean in separate analyses. We hypothesized that neonatal outcomes might be better among women with a pre-labor cesarean birth, consistent with prior literature [5].

## Methods

### Study design

Data analyzed represent that of a prospectively conducted study in communities in Chimaltenango, Guatemala from January 2017 through April 2020, through the Global Network for Women’s and Children’s Health Research, Maternal and Newborn Health Registry (MNHR) [6]. The MNHR is a population-based prospective registry of pregnancies at Global Network sites to provide data on pregnancy outcomes [6].

### Setting

There are 8 distinct clusters in the Guatemalan MNHR including those served by health posts (42), health centers (30), and a referral hospital [6]. Each community generally experiences between 300 and 500 deliveries annually [6]. The MNHR enrolls women as early as possible during pregnancy and collects pregnancy outcomes after birth [6].

### Population/Recruitment

Only women with a history of one prior cesarean delivery with data on mode of birth (timing of cesarean) of the index pregnancy were included. Data were excluded from women who were enrolled but medically terminated pregnancies (*n* = 12), experienced miscarriages (*n* = 566), died prior to labor and delivery (*n* = 7), were lost to follow-up prior to delivery (*n* = 7), or those with missing data for delivery mode (*n* = 1806). The study population included pregnant women who were eligible, consented, and delivered by repeat cesarean birth in the study period [6].

### Primary outcomes

The primary outcomes of this analysis were characteristics (sociodemographic, antepartum and obstetric, and maternal and perinatal outcomes) associated with timing of repeat cesarean birth (pre-labor versus intrapartum) among women with a history of prior cesarean.

### Secondary outcomes

The secondary outcomes were maternal and perinatal/neonatal outcomes associated with the timing of the repeat cesarean birth.

### Analysis plan

We used descriptive statistics to produce counts and percentages on timing of repeat cesarean among women with a history of prior cesarean delivery. Then we observed independent variables associated with timing of cesarean, and performed bivariate comparisons of sociodemographic and antenatal covariates, intrapartum characteristics, and maternal and perinatal/neonatal outcomes that we hypothesized might be associated with mode of delivery. *P*-values were obtained from bivariate comparisons as a function of each individual risk factor using Kruskal–Wallis, Fisher’s Exact, or Chi-squared tests depending on variable type.

All risk factors that occurred before delivery and might be associated with type of cesarean were included in a logistic regression (*p* < 0.05 from the individual risk factor bivariate comparisons). We then used individual logistic regressions with type of cesarean as the dependent variable with maternal and perinatal/neonatal outcomes that were significantly different in bivariate comparisons by type of cesarean (*p* < 0.05 from the individual risk factor bivariate comparisons). No methods were used to adjust for any potential bias. All data analyses were performed with STATA software v.15.1. (STATA Corp, College Station, TX, USA).

## Results

**Fig. 1:**
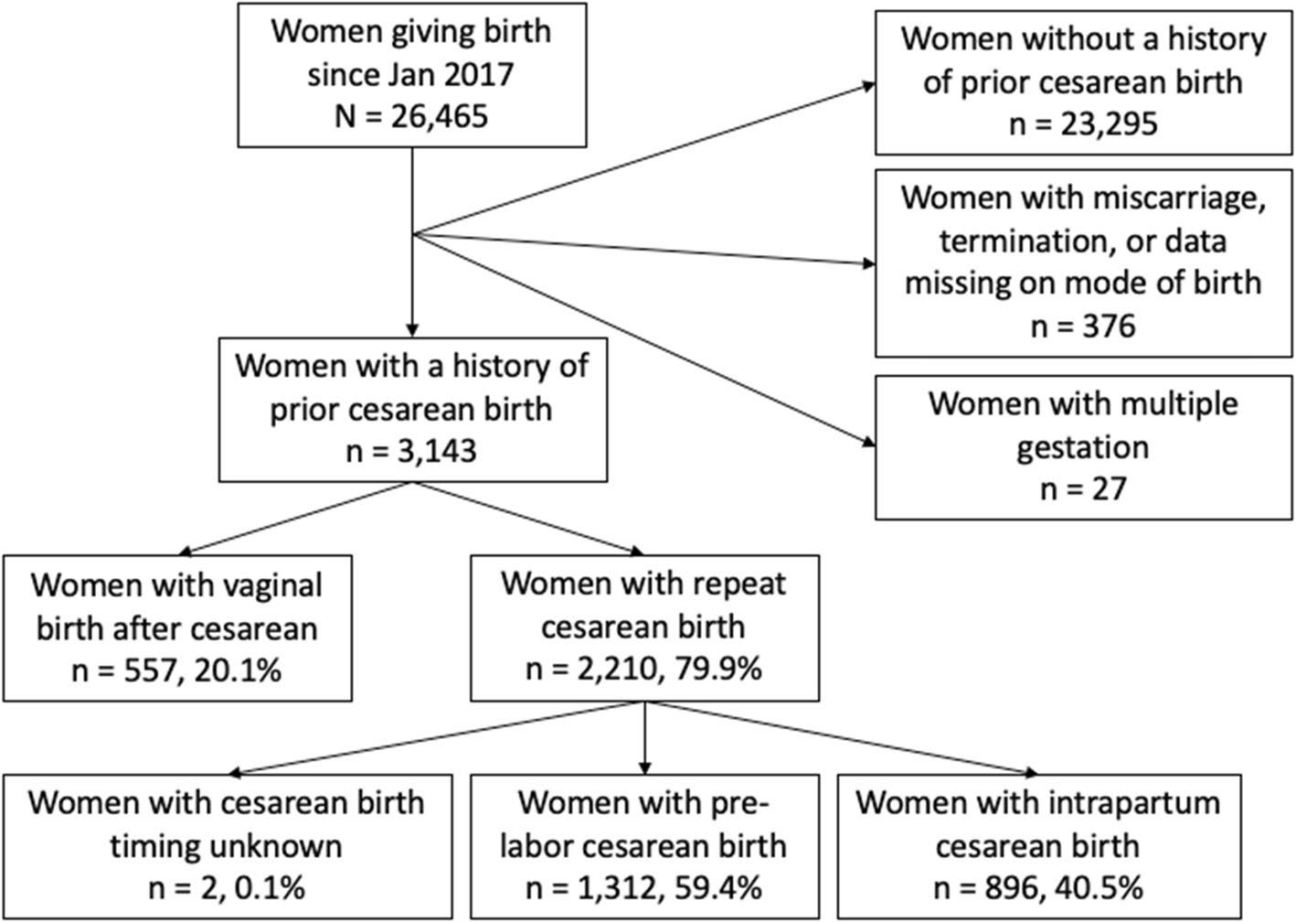
Population of women with a history of prior cesarean birth and mode of subsequent delivery at the Guatemalan site of the Global Network for Women’s and Children’s Health Research, Maternal and Newborn Health Registry

Figure 1 is a flow diagram of the population of women included in this study. Between January 2017 and April 2020, 26,465 women delivered in the Guatemalan clusters of the MNHR. 3,143 women, 11.9% of the MNHR population had a history of prior cesarean delivery and a singleton gestation. 2,210 (79.9%) women with a history of prior cesarean birth had data available on mode of delivery and gave birth by repeat cesarean; 1312 (59.4%) were pre-labor cesareans while 896 (40.5%) were intrapartum cesarean births. The remaining nearly 20% of women who achieved successful vaginal birth after cesarean are considered in a separate analysis and future research targeting the inequity in mode of birth is being pursued.

Table 1 presents the sociodemographic and obstetric/labor characteristics of the population overall and by timing of cesarean. The population median age was 27 with interquartile range (IQR) 23 to 31 years. Most women had schooling (95.8%; data on years of schooling was not available), more than half were primiparous (58.6%), and 71.0% were of overwight or obese body mass index (BMI). Women who delivered by intrapartum cesarean birth (as compared to pre-labor cesarean) were statistically more likely to be younger (median age 26 versus 27), less likely to have had schooling (94.0% versus 96.0%), and more likely to be underweight or normal weight (32.6% versus 26.7%), *p* $\le$ 0.05. The median interpregnancy interval of women experiencing intrapartum cesarean was shorter (26.3 versus 30.3 months) and they experienced less hypertensive disease (4.8% versus 8.4%), *p* < 0.05. For these same women, those undergoing intrapartum cesarean birth, they experienced more obstructed labor (7.2% versus 2.7%) and were more likely to deliver in the hospital (95.8% versus 91.1%) compared to “other” locations, *p* < 0.05.

**Table 1:** Population characteristics of women with a history of prior cesarean birth overall and by mode of delivery, January 2017 – April 2020

	Women giving birth by cesarean *N* = 2208	Pre-labor cesarean birth *n* = 1312, 59.4%	Intrapartum cesarean birth *n* = 896, 40.6%	*P*-value
*Sociodemographics*				
Age in years [IQR]	27 [23,31]	27 [23,31]	26 [22,30]	** < 0.001**^a^
Schooling n, %				0.05^b^
Illiterate Literate, no school Schooling	93, 4.2% 13, 0.6% 2102, 95.2%	44, 3.4% 8, 0.6% 1260, 96.0%	49, 5.5% 5, 0.5% 842, 94.0%	
Parity n, %				0.91^c^
1 2 3 +	1294, 58.6% 662, 30.0% 252, 11.4%	765, 58.3% 398, 30.3% 149, 11.4%	529, 59.0% 264, 29.5% 103, 11.5%	
BMI kg/m^2^				**0.004**^b^
< 18.5 18.5 – 24.9 25 – 29.9 $\ge$ 30	10, 0.4% 633, 28.6% 952, 43.2% 613, 27.8%	5, 0.4% 346, 26.3%% 565, 43.1% 396, 30.2%	5, 0.6% 287, 32.0% 387, 43.2% 217, 24.2%	
*Antepartum & obstetric characteristics*				
IPI in months [IQR]	29.1 [16.1,48.1]	30.3 [17.3,50.6]	26.3 [14.8,45.1]	** < 0.001**^a^
Antenatal care n, %	2172, 98.4%	1293, 98.6%	879, 98.1%	0.41^c^
Female sex of baby n, %	1120, 50.8%	663, 50.6%	457, 51.0%	0.84^c^
Missing	1, 0.1%	1, 0.1%	0, 0.0%	
Birthweight in grams [IQR]	2890 [2637,3147]	2892 [2640,3150]	2860 [2608, 3120]	0.13^a^
Missing	2, 0.1%	2, 0.1%	0, 0.0%	
Term gestational age n, %	2075, 94.0%	1232, 93.9%	843, 94.1%	0.86^c^
Obstructed labor n, %	101, 4.6%	36, 2.7%	65, 7.2%	** < 0.001**^**c**^
Antepartum hemorrhage n, %	5, 0.2%	2, 0.2%	3, 0.3%	0.40^b^
Hypertensive disease n, %	153, 6.9%	110, 8.4%	43, 4.8%	**0.001**^**c**^
Induction of labor n, %	22, 1.0%	10, 0.8%	12, 1.3%	0.18^c^
Referred in labor n, %				0.23^c^
Yes Missing	417, 18.9% 1, 0.04%	237, 18.1% 0, 0.0%	180, 20.1% 1, 0.1%	
Attendant n, %				0.18^c^
Non-OB MD OB	68, 3.1% 2140, 96.9%	35, 2.7% 1277, 97.3	33, 3.7% 863, 96.3%	
Delivery location n, %				** < 0.001**^c^
Hospital Other	2053, 93.0% 154, 7.0%	1195, 91.1% 116, 8.9%	858, 95.8% 38, 4.2%	

All tests performed excluding missing data

*IPI* interpregnancy interval

^a^ kruskall-wallis

^b^ fishers exact

^c^ chi^2^

Table 2 shows maternal and neonatal outcomes that varied in bivariate comparisons by timing of cesarean birth. Magnesium sulfate was administered to more women undergoing pre-labor cesarean (7.3% versus 4.2%), and the rate of postpartum infection was higher with pre-labor cesarean birth than intrapartum (0.5% versus 0.0%), *p* < 0.05. With respect to neonatal outcomes, none were statistically different between the two types of cesarean.

**Table 2:** Maternal and neonatal outcomes of women with a history of prior cesarean birth overall and by mode of delivery, January 2017 – April 2020

	Women giving birth by cesarean	Pre-labor cesarean birth	Intrapartum cesarean birth	*P*-value
	*N* = 2208	*n* = 1312, 59.4%	*n* = 896, 40.6%	
*Maternal outcomes*				
Hemorrhage n, %	15, 0.7%	7 0.5%	8, 0.9%	0.43^b^
Uterotonics n, %	2166, 98.1%	1282, 97.8%	884, 98.7%	0.14^c^
Blood transfusion n, %	24, 1.1%	13, 1.0%	11, 1.2%	0.68^b^
D&C/Suction n, %	3, 0.1%	3, 0.2%	0, 0.0%	0.28^b^
Magnesium n, %	134, 6.1%	96, 7.3%	38, 4.2%	**0.003**^**c**^
Hysterectomy n, %	12, 0.5%	8, 0.6%	4, 0.4%	0.77^b^
Severe infection n, %	32, 1.4%	19, 1.4%	13, 1.4%	1.0^c^
Postpartum infection n, %	7, 0.3%	7, 0.5%	0, 0.0%	**0.046**^**b**^
Missing	68, 3.1%	32, 2.4%	36, 4.0%	
Seizure n, %	3, 0.1%	1, 0.1%	2, 0.2%	0.57^b^
Missing	68, 3.1%	32, 2.4%	36, 4.0%	
Unplanned hospitalization n, %	17, 0.8%	12, 0.9%	5, 0.5%	0.46^b^
Missing	68, 3.1%	32, 2.4%	36, 4.0%	
*Neonatal outcomes*				
Fetal status n, %				0.17^b^
Born alive, alive	2180, 98.8%	1290, 98.4%	890, 99.3%	
Born alive, neonatal demise	5, 0.2%	4, 0.3%	1, 0.1%	
Stillbirth	22, 1.0%	17, 1.3%	5, 0.6%	
Bag & mask resuscitation n, %	22, 1.0%	12, 0.9%	10, 1.1%	0.64^c^
Missing	1, 0.1%	1, 0.1%	0, 0.0%	
Breastfed within an hour n, %	135, 6.2%	85, 6.6%	50, 5.6%	0.36^c^
Neonatal antibiotics n, %	91, 4.1%	50, 3.8%	41, 4.6%	0.38^c^
CPAP n, %	6, 0.3%	6, 0.5%	0, 0.0%	0.09^b^
Missing	1, 0.1%	0, 0.0%	1, 0.1%	
Oxygen n, %	101, 4.6%	54, 4.1%	47, 5.3%	0.21^c^
Ventilation n, %	11, 0.5%	7, 0.5%	4, 0.5%	1.0^b^
Missing	1, 0.1%	0, 0.0%	1, 0.1%	
Death by 42 Days n, %	28, 1.3%	16, 1.2%	12, 1.3%	0.79^c^
Missing	95, 4.3%	53, 4.0%	42, 4.7%	

All tests performed excluding missing data

*CPAP* continuous positive airway pressure

^a^kruskall-wallis

^b^fishers exact

^c^chi^2^

Table 3A shows multivariable modeling of timing of cesarean including all variables occurring prior to delivery significant in bivariate comparisons (delivery location, hypertensive disorders of pregnancy, labor dysfunction, interpregnancy interval, BMI, education, and age). The table shows variables that were associated with intrapartum cesarean birth when compared to pre-labor cesarean birth. Those associated with an increased risk of intrapartum cesarean birth included hospital delivery as compared to “other” location (ARR 1.6 [1.2,2.1]) and dysfunctional labor (ARR 1.6 [1.4,1.9]). Variables associated with a reduced risk of intrapartum cesarean birth were hypertensive disease (ARR 0.7 [0.6,0.9]), schooling (ARR 0.9 [0.8,0.9]), and increasing age, which was associated with a very slight reduction in the outcome (ARR 0.99 [0.98,0.99]).

**Table 3:** Intrapartum cesarean birth compared with pre-labor cesarean birth

Characteristic	RR	95% CI	*P*-Value
*(A) Multivariable poisson model with robust error variance of characteristics associated with intrapartum cesarean birth compared to pre-labor cesarean birth*^a^			
Hospital delivery (ref: “other” delivery location)	1.6	1.2,2.1	**0.001**
Hypertensive disease (ref: no hypertensive disease)	0.7	0.6, 0.9	**0.01**
Dysfunctional labor (ref: no labor dysfunction)	1.6	1.4,1.9	** < 0.001**
Schooling (ref: no formal schooling, illiterate)	0.9	0.8,0.9	**0.009**
Increasing age (continuous variable)	0.99	0.98, 0.99	**0.02**
*(B) Individual multivariable poisson models with robust error variance, adjusted for significant findings in Table *3*a , to determine association of intrapartum cesarean birth (with reference to pre-labor cesarean birth) with outcomes significant in bivariate comparisons*^b^			
Maternal outcomes			
RR of postpartum infection	Model did not converge because no intrapartum cesarean births were followed by postpartum infection (see Table 2)		
RR of needing magnesium sulfate	1.0	0.9,1.1	0.99

^a^ Bivariate comparisons of characteristics with *p* < 0.05 for intrapartum cesarean compared to vaginal birth after cesarean that were included in multivariable model (3A): delivery location, hypertensive disorders of pregnancy, labor dysfunction, interpregnancy interval, body mass index, education, age

^b^ Bivariate comparisons of maternal and neonatal outcomes with *p* < 0.05 for intrapartum cesarean compared to vaginal birth after cesarean that were tested in multivariable model (3B): maternal outcomes: postpartum infection, magnesium; neonatal outcomes: neonatal outcomes did not differ by timing of cesarean birth

Table 3B shows the significant results of individual regressions were timing of cesarean was tested as the dependent variable with the maternal outcomes that differed in bivariate comparisons as the indepent variable. These outcomes included postpartum infection and magnesium. Each regression was adjusted for delivery location, hypertensive disease, dysfunctional labor, education, and age. Postpartum infection did not occur after intrapartum cesarean birth, so the model did not converge, and the adjusted relative risk of needing magnesium did not vary by timing of cesarean birth. No neonatal outcomes were tested as none were significant in bivariate comparisons.

## Discussion

The main findings of this analysis were that women with a history of prior cesarean with hypertensive disease, more education, or older age were more likely to pursue pre-labor repeat cesarean birth, while women experiencing dysfunctional labor or those delivering in the hospital were more likely to undergo repeat cesarean birth during the course of labor. Notably, maternal and perinatal outcomes did not vary by timing of the repeat cesarean birth. This data is from a large Guatemalan cohort and may be generalizable to similar Latin American populations.

Our findings regarding hypertensive disease, age, and higher education being associated with pre-labor repeat cesarean birth in this cohort of Guatemalan women are consistent with the literature regarding cesarean birth, generally [7]. Hypertensive disease (reflected by magnesium sulfate findings as well), while not an absolute indication for cesarean birth, can often be treated with delivery, which may be intentionally expedited by cesarean birth [8, 9]. Similarly, as women age or have higher levels of education, they are often more likely to seek and/or undergo cesarean birth, which applied to this pre-labor repeat cesarean cohort, as well [10]. Therefore, these findings are not novel and are consistent with prior literature.

Regarding risk factors that reduced the likelihood of pre-labor repeat cesarean birth, these were delivery in the hospital and dysfunctional labor. Only women attempting labor are eligible to experience dysfunctional labor, so it is not unexpected that this obstetric characteristic was associated with intrapartum repeat cesarean birth. Additionally, this is a known risk factor for cesarean birth, generally [11]. Our delivery location result is hard to interpret but may represent the difference in cesarean birth that are commonly seen when comparing public to private hospitals [12]. It is known that many of the women in this cohort deliver in a public hospital, so it is possible that the ‘other’ delivery location represents a private facility. Private facilities have higher cesarean birth rates and more likelihood of elective cesarean birth, which is analogous to pre-labor cesarean birth in the case of women with a history of prior cesarean [13].

An unexpected finding was the rate of postpartum infection being higher with pre-labor cesarean than intrapartum. Usually, postpartum infection occurs more commonly after an intrapartum course than after an elective cesarean [9]. It is a limitation that we are not comparing the cesarean birth group to the vaginal birth after cesarean group in this particular analysis, because understanding the postpartum infection rate in that population may give a reference point for interpretation. We do not have a hypothesis for this finding but note it as a potential area for future research.

Strengths of the analysis are the large sample size, which contributes to external validity, the high quality of the data, and the breadth of antepartum, intrapartum, and postpartum variables that were included in the analysis. This analysis is limited in lacking data on the preferred mode of delivery of these women with a history of prior cesarean. For example, we do not know if the intrapartum cesarean births represent women who desired a trial of labor after cesarean or those who desired a pre-labor cesarean birth but presented to the facility in spontaneous labor. Additionally, we do not have data from observation or chart review or survey about indications for cesarean birth and potentially how contraindications or other patient or labor characteristics may have contributed to mode of birth. Details on indication for cesarean are being added to the dataset so this would allow for additional future secondary analyses on this and related topics.

## Conclusion

In conclusion, this analysis found that maternal and neonatal outcomes are no worse after intrapartum cesarean births compared to pre-labor cesarean births, supporting the null hypothesis. While this result is only one consideration in a highly complex decision-making process regarding mode of delivery after a prior cesarean, these results may help in the counseling of women regarding the risks and benefits of pre-labor cesarean birth versus attempting trial of labor after cesarean, which may result in an intrapartum cesarean birth. Studying outcomes by women’s preferred delivery mode would be an important area for future research.

## Data Availability

The data that support the findings of this study are available from the National Institutes of Health Data and Specimen Hub, but restrictions apply to the availability of these data, which were used under license for the current study, and so are not publicly available. The Data and Specimen Hub should be contacted for access to the data at the following address: https://dash.nichd.nih.gov/study/20225.

## Abbreviations

WHO: World Health Organization
MNHR: Global Network for Women’s and Chilren’s Health Research Maternal and Newborn Health Registry
IQR: Interquartile range
BMI: Body mass index
ARR: Adjusted relative risk
MNHR: Maternal and newborn health registry
